# Bladder paraganglioma: a case report

**DOI:** 10.11604/pamj.2020.36.339.23086

**Published:** 2020-08-25

**Authors:** Gil Falcão, Cabrita Carneiro, Luís Campos Pinheiro

**Affiliations:** 1 Serviço de Urologia, Centro Hospitalar Universitário de Lisboa Central, Lisboa, Portugal

**Keywords:** Bladder paraganglioma, bladder tumor, micturition syncope, transurethral bladder resection, cystectomy

## Abstract

Bladder Paraganglioma is a rare type of bladder tumor (0.06%). It is typically benign and the most common symptoms are hematuria, hypertension and headache. About 14% of these tumors are malign and consequently radio and chemoresistants. Therefore, surgery is the mainstay of treatment. As they are likely to recur and to metastize lifelong follow-up is required. The authors report a rare case of a 53 years old man with hematuria and a previous history of micturition syncope who was diagnosed with bladder lesion. During the transurethral ressection of bladder he became severely hypertensive. Plasma metanephrines, and urinary vanillylmandelic acid, were still high and the exams suggested residual tumor. The patient underwent radical cistoprostatectomy. After 4 years of follow-up the patient remains disease free.

## Introduction

Approximately 10% of pheocromocytomas occur at extra-adrenal sites [1], with <0, z05% accounting for bladder paragangliomas (BPG) [2]. These tumors are typically benign, and the most common symptoms are hypertension, headache, hematuria and palpitations, with characteristic micturition attacks. Complete surgical resection of the tumour through transurethral ressection of bladder tumor (TURBT) or partial cistectomy can be curative in many cases. Life-long clinical and biological follow-up of patients is essential as dissemination or local recurrence of malignant BPG can occur very late in the clinical course following removal of the tumour. Due to their rarity, there are few published studies on the diagnostic evaluation and therapeutic orientation of these patients. We describe a case of a patient with BPG considering the clinical presentation, imaging findings and treatment options.

## Patient and observation

**Figure 1:**
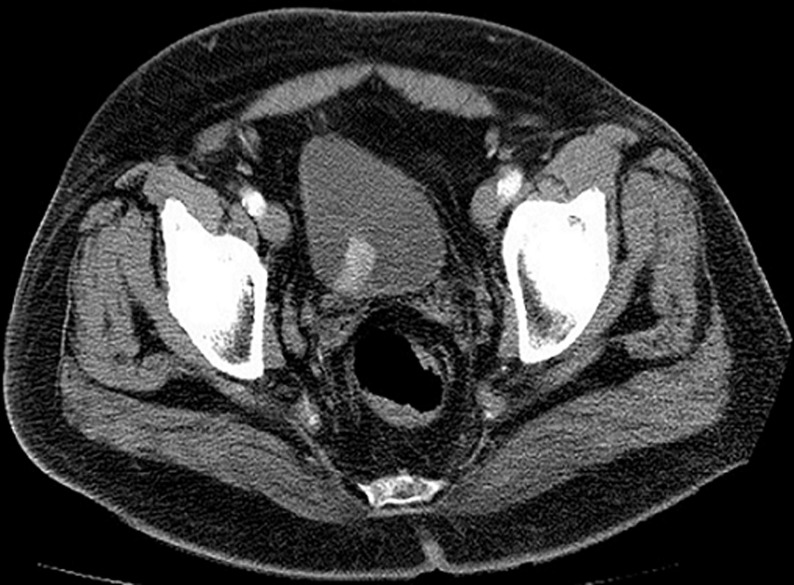
CT scan showing bladder lesion

**Figure 2:**
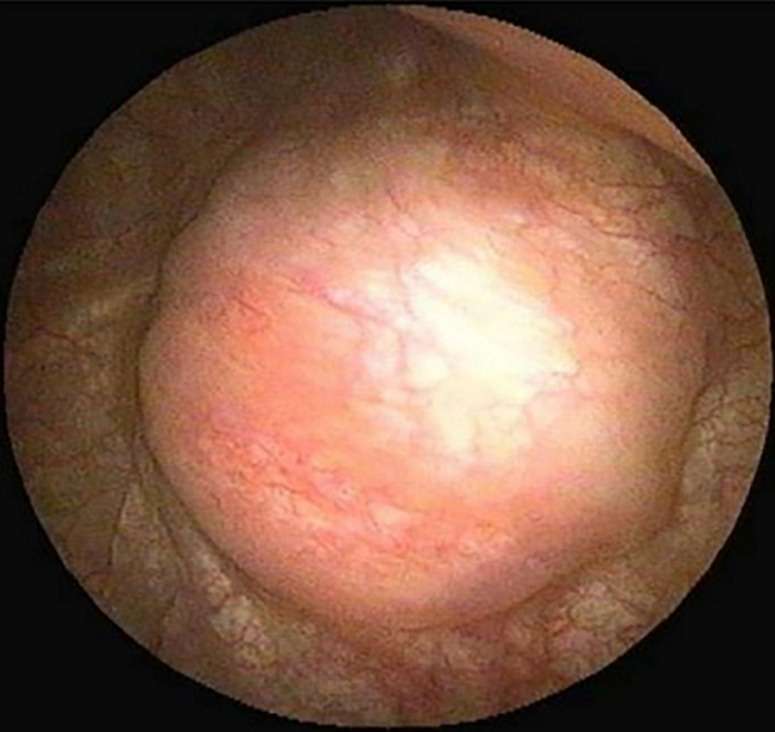
cystocopy showing bladder mass on the lateral right wall

**Figure 3:**
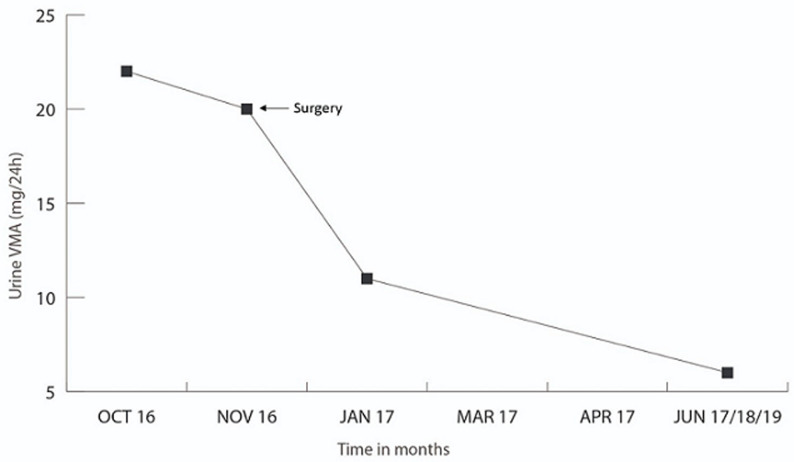
urinary vanillylmandelic acid levels before and after surgery

History of a previously healthy 53 years old male patient with a history of 3 episodes of painless gross hematuria, and one micturition syncope. A contrasted-enhanced CT scan (CECT) was performed and there was a contrast enhanced bladder mass with full wall invasion (Figure 1). No lymph nodes or peripheral lesions were detected. In October 2016, during the TURBT (Figure 2) procedure he became severely hypertensive but controled under antihypertensive drugs. Histological examination of the TURBT specimen showed presence of a BPG. At 1-month postoperatively, plasma metanephrines (PM), and urinary vanillylmandelic acid (VMA), were still high and the CECT scan plus cistoscopy suggested residual tumor. The patient underwent radical cistoprostatectomy in December 2016. Histological examination showed a BPG. The 24h urine showed a dramatic drop in VMA level after the surgery until now (Figure 3). After 4 years of follow-up with cistoscopy, CECT and VMA levels, the patient remains disease free.

## Discussion

Pheochromocytomas are catecholamine-secreting tumors derived from adrenal glands and the sympathetic nervous system. Extra-adrenal pheochromocytomas, known as paragangliomas, account for about 15% of all pheochromocytomas [3]. Germ line mutations in B, C, and D subunit- coding genes of the succinate dehydrogenase mitochondrial Complex II are associated with multiple paragangliomas [4]. BPG is a rare tumor that was first described by Zimmerman in 1953 and belongs to nonurothelial bladder tumors. The classic triad of clinical symptoms is silent macroscopic hematuria (present in 60% of reported cases), paroxysmal hypertension, and “urinary attacks”. They are most commonly situated at the dome or the trigone of the bladder and the position of these tumours within the bladder cause a characteristic symptom complex most commonly related to micturition or over-distension of the bladder causing catecholamine release. Pre-operative diagnosis is usually suspected on the grounds of the typical clinical symptoms and signs mentioned and further confirmed by measurement of biogenic amines in both urine and plasma [5]. Both CT scanning and MRI are useful in the localisation of both the primary tumour and any metastases. However, scanning with Iodine metaiodinebenzylguinidine (MIBG) has a very high sensitivity and specificity for phaeochromocytoma detection. Given the relatively low incidence of malignant bladder paragangliomas, complete surgical resection of the tumour can be curative in many cases [6]. Many experts advocate complete surgical removal as the standard management for paragangliomas, including partial cystectomy or radical cystectomy, because it originates from the intra-mural portion of the bladder wall (therefore it is difficult to remove the tumor entirely with TURBT only). However, some surgeons still perform TURBT and follow the patients regularly for small tumors at special tumor locations. Radiotherapy and chemotherapy have shown limited effectiveness in the treatment of locally recurrent and metastatic paraganglioma.

Histologically there are no definitive characteristics which reliably distinguish benign from malignant tumours. The most well-known pathological finding refers to the “Zellballen” pattern, in which immunohistochemical staining is usually positive for synaptophysin and chromogranin, with S-100 being highlighted in sustentacular cells. A review of studies looking at other factors (including biochemical and genetic markers) for determination of malignancy by Pattarino *et al*., came to the conclusion that metastatic dissemination is the only real proof of malignancy [7]. Pre-operatively the patient requires α-adrenergic blockade and volume expansion as these measures have been shown to significantly reduce peri-operative mortality and morbidity [8]. The interesting feature of the case presented above is that the patient reported one of the micturition-induced symptoms of catecholamine release typical of primary BPG - syncope. During the TURBT he suffered a hypertensive crisis that was controlled with α-blocker. Tsai *et al*. suggested that post-operative follow-up protocols should include annual cystoscopy, plasma or urine catecholamine analysis and an MIBG scan, while Young *et al*., regarded anual urine and serum VMA to be the best tools for detecting clinical recurrence or distant metastasis [9]. Like few cases reported in the literature, there was tumor recorrence after the initial TURBT and the biogenic amines were still high as well. Ranaweera [10] reported three cases of BPG, very similar to the one we describe. Two cases were sorted out only with TURBT and the last one underwent partial cystectomy with ureteral reimplantation. All patients had a similar follow-up with serum and urinary catecholamines, cystoscopy and CT scan. The authors decided to perform a radical cystectomy and four years later the patient is disease free as confirmed by CECT, cystoscopy (through the ileal conduit) and VMA levels (every 6 months), which are normal. Life-long clinical and biological follow-up of patients is essential as dissemination or local recurrence of malignant paragangliomas can occur very late in the clinical course following removal of the tumour.

## Conclusion

Bladder paraganglioma is a rare condition and patients can present with various clinical presentations. Young age, extensive local disease and micturition attacks are risk factors for malignancy while features such as vascular invasion, a deeply invasive growth patterns and recurrence are often poor prognostic signs. Like our study shows, malignant phaeochromocytoma remains a challenging entity to diagnose and treat. Surgical extirpation remains the only cure for these patients and further research into this rare condition is warranted. This case highlights one of the clinical presentation of bladder paraganglioma and provides a clinical review of the current literature on management of this rare condition. Due to its rarety we would like to add our experience to the few cases published so far.
